# Spent Mushroom Substrate and Electric Arc Furnace Dust Recycling by Carbothermic Reduction Method

**DOI:** 10.3390/ma15072639

**Published:** 2022-04-03

**Authors:** Hao-Hsun Chang, In-Gann Chen, Hao-Yun Yu, Meng-Yu Tsai, Keng-Tung Wu, Shih-Hsien Liu

**Affiliations:** 1 Department of Materials Science and Engineering, National Cheng Kung University, Tainan 701, Taiwan; n58041036@gs.ncku.edu.tw (H.-H.C.); o1gogo123@gmail.com (H.-Y.Y.); j6986422@hotmail.com (M.-Y.T.); 2 Department of Forestry, National Chung Hsing University, Taichung 402, Taiwan; wukt@nchu.edu.tw; 3 China Steel Corporation, Kaohsiung 812, Taiwan; 124552@mail.csc.com.tw

**Keywords:** agricultural waste, spent mushroom substrate, electric-arc furnace dust, carbothermic reduction, carbon neutralization, circular economy

## Abstract

With recent increases in environmental awareness, the circular economy concept, which involves turning waste into usable products, has gradually become widely accepted. Spent mushroom substrate (SMS) is an agricultural waste that lacks recycling channels in Taiwan. This study explored the feasibility of simultaneously recycling two completely different types of waste: spent mushroom substrate (SMS), an agricultural waste, and electric-arc furnace dust (EAFD), an industrial waste. Specifically, SMS was used to replace metallurgical coke as a reducing agent for EAFD, which underwent carbothermic reduction to recycle valuable metallic Zn. The results showed that if SMS and EAFD were mixed at a C/O ratio of 0.8, the degree of Zn removal achieved 95% at 1100 °C, which is 150 °C lower than the reduction temperature of the EAFD-coke mixture (due to volatile matter (VM) in SMS). For the reduction of ZnO in EAFD, with the assistance of VM in SMS, the C/O ratio can be decreased from 0.8 to 0.16 at 1300 °C, achieving a high degree of Zn removal over 95%. In addition, the torrefaction of SMS increased the fixed carbon content and improved the Zn productivity at the same C/O ratio, reaching almost the same productivity as the coke sample (SMS torrefaction = 500 °C, C/O = 0.8, reduction = 1200 °C, Zn removal~99%). Finally, CO_2_ emission reductions from the use of SMS were also estimated.

## 1. Introduction

Spent mushroom substrate (SMS) is a type of agricultural waste that is difficult to transport due to its bulk and high water content. Taking Taiwan as an example, the annual output of SMS exceeds 300,000 tons [1], even though these agricultural residues can be mostly transformed into compost materials [2]. The use of plastic bags as packaging and a variety of fertilizers in the process of mushroom farming leads to the presence of chlorine in SMS agricultural waste; therefore, incineration is not a desirable disposal method because it produces PM 2.5 and the crucial carcinogenic dioxin. Furthermore, stringent governmental regulations for dioxins and furans produced by waste-to-energy plants (dioxin and furans <0.1 ng-TEQ/Nm^3^) make SMS unable to be disposed of by incineration under Taiwan’s regulations. As a result, SMS disposal is often landfilled or composted, which incurs disposal costs and leads to land contamination. 

According to the waste management hierarchy, “Reuse” and “Recycle” are more beneficial than “Recover” (including incineration) to the environment. Researchers had investigated methods for partial recovery of SMS, including its conversion back into horticultural soil through nutrient addition [3]. Several studies had investigated the feasibility of converting SMS into fuel for power generation after granulation [4,5]. The calorific values vary from 12.1 MJ/kg to 13.7 MJ/kg for the dry base SMS [6]. As the torrefaction temperature increased, the high heating value (HHV) gradually increased [7]. In addition, the conversion of SMS to biogas had also been studied [8,9]. Researchers also demonstrated that SMS biochar was a potential candidate for the efficient removal of heavy metals [10]. Another method to increase the added value of SMS recovery is to use its carbonaceous properties as a reducing agent for carbothermic reduction to replace high-priced metallurgical coke, but the current research is relatively insufficient. Therefore, this study utilized SMS as a reducing agent to create added value and disposed of SMS waste in an economical and environmentally friendly manner in line with the concept of circular economy. An electric-arc furnace (EAF) is a furnace that melts and recycles scrap steel, in the process producing electric-arc furnace dust (EAFD), an industrial waste [11]. The composition of EAFD depends on the type of scrap used and other additives, and may include magnetite (Fe_3_O_4_), franklinite (ZnFe_2_O_4_), and other elements at lower concentrations, such as Cl, Pb, and Cr. Lead and chromium are considered threats to human and environmental health [12,13]. Chlorine had been reported to account for approximately 5% of EAFD as a result of increased Cl-containing impurities in scrap steel such as paints [14]. Therefore, EAFD is generally classified as a toxic waste requiring special handling. 

In Taiwan, the Waelz process is commonly used to process EAFD by carbothermic reduction [15]. Specifically, fossil fuels such as metallurgical coal and/or coke serve as reducing agents, and CaO and SiO_2_ serve as additives for the reduction of a non-ferrous metal, Zn, in EAFD. Oxygen is blown in at the discharge end of the kilns. The gases exiting the kilns containing non-ferrous metals are cooled and passed through filtering equipment. The reduced Zn is released in gaseous form and subsequently converted to ZnO powder through reoxidation at the end of the process. The remaining FeOx and other elements become slag. However, using coal and coke as reductants in carbothermic reduction generates massive amounts of CO_2_, which is inconsistent with global emissions reduction targets, and may incur substantial costs due to carbon taxes. 

Alternatively, using biomass to replace or partially replace coal and coke can reduce CO_2_ emissions and potentially improve the process efficiency [16,17]. Others had used charcoal and petroleum coke for the carbothermic reduction of EAFD and had reported higher compressive strength and improved reduction of charcoal containing pellets [18]. In these studies, the reaction temperature was the most critical determinant of the reduction rate.

Therefore, if SMS can successfully replace coke as the reducing agent for EAFD, the economic value of recycled SMS can be expected to increase substantially. According to China Coke Spot Price, as of 15 February 2022, the price of metallurgical coke was approximately 400 USD per ton (average 310 USD per ton in the past ten years), whereas that of biofuel was approximately 150 USD per ton.

In addition, the high temperature process can prevent the production of dioxins, because under anoxic (oxygen-deficient) conditions, dioxin starts to decompose at 300–500 °C, while at 800 °C, dioxin can be completely decomposed within 21 s [19]. Therefore, the impact on the environment can also be reduced. Moreover, the use of SMS (biomass) as a reducing agent can reduce net CO_2_ emissions. However, SMS has high water content, low energy density, low heat value, poor uniformity, and is difficult to grind.

Nevertheless, these shortcomings can be compensated by torrefaction, which had been reported to significantly improve the combustion, gas production, thermal conversion efficiency, and energy density of SMS [20]. Furthermore, increasing the temperature of torrefaction had been reported to increase the fixed carbon content and specific energy density of biomass carbon [21]. 

In view of the published literature described above, there are few studies on the effect of volatile matter in biomass on zinc oxide reduction. Therefore, by comparing the differences in torrefaction temperature and fixed carbon molar ratio between SMS and coke, the effect of volatiles was revealed in this study. By simultaneously processing wastes from two completely different industries (electric furnace steelmaking and mushroom farming), we propose a different approach to enhancing economic incentives and net CO_2_ emissions. Therefore, we investigated the feasibility of using SMS (instead of coke) as a reducing agent for EAFD.

## 2. Materials and Methods

### 2.1. Raw Materials

Initially, the moisture content of SMS is about ~70%. SMS were dehydrated followed by torrefaction for 1 h at 300 °C or 500 °C in nitrogen environment, respectively. Table 1 shows the approximate analytic results using metallurgical-grade coke as a reference to current industrial conditions. A series of three types of SMS (i.e., without torrefaction, torrefied at 300 and 500 °C, respectively) were used in this study. The results showed that the fixed carbon in SMS increased from 17.9% (un-torrefied) to 59.0% (torrefied at 500 °C) with the increase of torrefaction temperature but was still lower than 86.9% of coke. Furthermore, the ash content of SMS also increased with increasing temperature. In contrast, volatile matter (VM) of SMS decreased from 72.5% (un-torrefied) to 39.0% (torrefied at 300 °C) and 20.3% (torrefied at 500 °C), respectively. The ash composition of SMS and coke was presented in Table 2. The main component of SMS ash was CaO, containing 43.2 mass %. In contrast, the ash in coke was mainly composed of SiO_2_ (56.3 mass %) and Al_2_O_3_ (25.2 mass %). In addition, it was worth mentioning that the content of P and K elements in SMS was higher than coke.

The EAFD used in this study was provided by China Steel Corporation (Taiwan). Table 3 showed its composition, mainly Fe_3_O_4_ (29.1 mass %) and ZnO (32.5 mass %). However, the composition of EAFD is complex and depends on the source of its waste. Notably, an increase in Cl-containing impurities such as rubber, paint, and polymers in scrap steel may lead to an increase in chlorine content in EAFD. As described by Pickles et al. [22], in the presence of sufficient chlorine, a chlorination reaction for lead, zinc, iron, calcium oxide, and zinc ferrite will be involved. The focus of this study is the recovery of the main components Fe_3_O_4_ and ZnO.

### 2.2. Carbothermic Reduction Experiment

The raw materials used in this experiment were ground and sieved with an 80-mesh sieve to ensure that the particle size did not exceed 180 μm. The EAFD and the reducing agent were mixed thoroughly, 1 mass % of bentonite (as a binder) and a small amount of deionized water was added, and the whole mixture was made into round sample pellets with a diameter of 15–16 mm.

After a day of air drying, the pellets were baked in an oven at 105–110 °C to remove moisture. Three series of samples with different fixed carbon-to-oxygen molar ratios (referred to as C/O ratios) were 0.16, 0.5, and 0.8, respectively. Based on these C/O ratios, proportional amounts of reducing agent and EAFD were prepared for these three series of sample pellets. As shown in Table 4, at the same C/O ratio, the ED-M sample had approximately twice the reducing agent percentage than the other samples, such as ED-MT3, ED-MT5, and ED-C. In other words, the ED-M sample had a lower EAFD percentage than the other samples, which will affect the amount of Zn and Fe recovered per gram of sample pellets, as will be discussed later in Section 3.4. This percentage difference was because the un-torrified SMS contained more than 70% VM (as shown in Table 1), the lower density of VM compared to EAFD would result in ED-M sample pellet weighing 2.8–3.7 g. This was significantly lower than the weight of 4.8–5.8 g of the other samples.

Heating was performed using a high temperature tube furnace (Lindberg/Blue M STF54434C). The sample pellet was placed on an alumina platform and then pushed into the furnace using a rod made of alumina for carbothermic reduction. During the carbothermic reduction, nitrogen gas was introduced at a rate of 3 L/min to prevent re-oxidation. The temperature was increased at a rate of 20 °C per minute until a temperature of 1300 °C was reached. The total reaction time was 60 min. At the end of the reaction, the reduced sample was moved to the furnace inlet to cool. To gain further insight into the carbothermic reduction of EAFD, some experiments were interrupted at temperatures of 600 °C, 800 °C, 900 °C, 1000 °C, 1100 °C, and 1200 °C, respectively. After 1 min of reaching these temperatures, the interrupted samples were moved to the furnace inlet, allowed to cool, and finally removed for analysis.

### 2.3. Analysis

**Figure 1:**
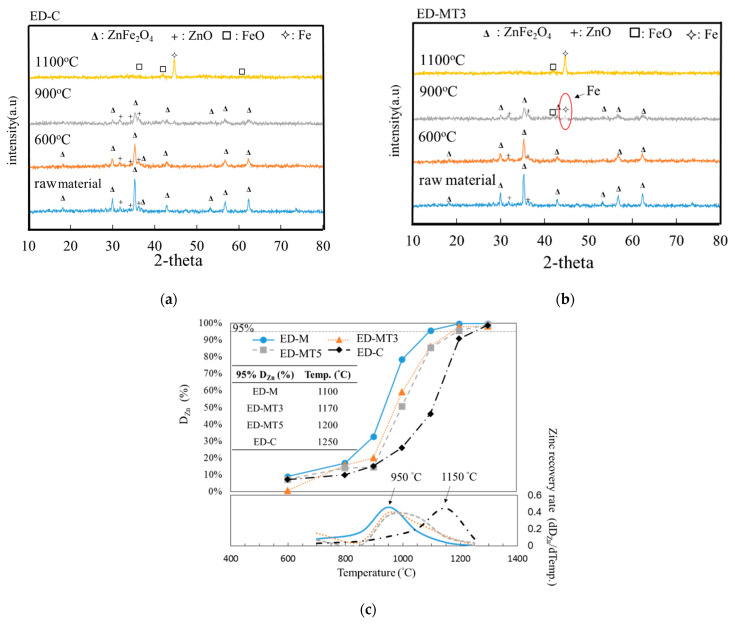
Carbothermic reduction of EAFD/SMS (ED-MT3) and EAFD/Coke (ED-C) composite samples with molar C/O = 0.8. (**a**) XRD patterns of ED-C samples interrupted at 600 °C, 900 °C, and 1100 °C during reduction process; (**b**) XRD patterns of ED-MT3 samples interrupted at 600 °C, 900 °C, and 1100 °C during the reduction process; (**c**) The upper curves showed the degree of zinc removal (%) defined in Equation (1) and the lower curves showed zinc recovery rate (dD_Zn_%/dTemp.). The recovery rate is defined as the derivative of upper curve, which is the derivative of the degree of removal with respect to temperature. The results show that the EAFD/SMS composite samples have the maximum recovery rate at about 950 °C, while the EAFD/C sample is about 1150 °C. This observation quantitatively suggests that SMS as a reducing agent (in place of coke) can lower the reduction temperature by about 200 °C.

The reduced samples were dissolved in acid and then analyzed for Zn content before, during (interrupted), and after carbothermic reduction using a high-resolution inductively coupled plasma-mass spectrometer (BRUKER Compact Q/TOF LC-MS System). The values thus obtained were then used to calculate the degree of Zn removal and Zn recovery rate shown in Figure 1c. The Zn content of the original and interrupted samples was calculated using the following equation:

(1) $$\mathrm{Degree}\;\mathrm{of}\;\mathrm{Zn}\;\mathrm{removal}\;(D_{\mathrm{Zn}},\;\%)=(W_{\mathrm{iZn}}-W_{\mathrm{fZn}})/W_{\mathrm{iZn}}\times 100\%$$

where W_iZn_ is the weight (g) of Zn in the original samples and W_fZn_ is the weight (g) of Zn in the reduced (or interrupted) sample and (W_iZn_-W_fZn_) is the weight loss after the reduction process.

In order to determine the critical temperature at which the carbothermic reduction occurs, Zn recovery rate at different temperatures was investigated. The Zn recovery rate is defined as the weight of Zn recovered per gram of sample pellet over a specific period of time (i.e., dD_Zn/_d time). Since the experiments were performed at a constant heating rate of 20 °C per minute, therefore, the Zn recovery rate can be defined as the derivative of the degree of Zn removal with respect to the temperature (dD_Zn/_dTemp.). 

To determine the degree of reduction of Fe_3_O_4_ in the samples at different temperatures, the metallic Fe content and total Fe content in the interrupted samples were performed by the wet chemical method. The degree of Fe metallization in the interrupted samples is shown in Section 3.4 and calculated using the following equation:

(2) $$\mathrm{Degree}\;\mathrm{of}\;\mathrm{Fe}\;\mathrm{metallization}\;(D_{\mathrm{Fe}},\;\%)=M.\;\mathrm{Fe}/T.\mathrm{Fe}\times 100\%$$

where M. Fe is the metallization degree of the composite sample and T. Fe is the total Fe of the sample.

The phase composition of the reduced samples was characterized using an X-ray diffractometer (Bruker AXS Gmbh/D2 Phaser) with the following settings: 2θ = 10–80°.; scan rate of 3 degrees/min; and scan resolution of 0.01 degrees/step. The change in average pore size during reduction process was measured using a specific surface area and porosimetry analyzer (Micromeritics Gemin 2360 BET-N2).

## 3. Results

### 3.1. Carbothermic Reduction of EAF Dust/Spent Mushroom Substrate Composite Pellets 

The compressive strength of the pellets was 2.4 to 2.6 Kgf/cm^2^ before the heating. The difference between ED-M, ED-MT3, ED-MT5, and ED-C is not significant. However, the compressive strength of the ED-M was 29.2 Kgf/cm^2^ lower than in another sample (ED-MT3: 70.7 Kgf/cm^2^, ED-MT5: 71.2 Kgf/cm^2^, and ED-C: 79.3 Kgf/cm^2^). In order to compare the difference between SMS-based reducing agents and coke, it is necessary to deeply study the reduction mechanism of EAFD. Figure 1a showed the x-ray diffraction (XRD) results of ED-C (EAFD + coke) sample at a C/O ratio of 0.8, which was subjected to carbothermic reduction at a heating rate of 20 °C/minute with interrupted at different temperatures, e.g., 600 °C, 900 °C, and 1100 °C, followed by cooling. Figure 1b showed a similar x-ray diffraction result of ED-MT3 (EAFD + SMS torrefied at 300 °C). Figure 1a showed that the phase composition of ED-C raw material sample is primarily Franklinite (ZnFe_2_O_4_ and ZnO); the XRD peaks of ZnO are relatively weak. At 600 °C, the XRD peaks of Franklinite phase weaken. At 900 °C, the peak intensity of ZnFe_2_O_4_ is decreased considerably, and the peaks of FeO become more pronounced. At 1100 °C, the XRD peaks of both FeO and metallic Fe become dominant phases.

Figure 1a,b have similar XRD patterns at 600 °C, where the Franklinite peaks dominate. By comparing the Figure 1a and Figure 1b, interestingly, at 900 °C, a Fe peak can be observed in the ED-MT3 sample. At 1100 °C, the Fe peak becomes a more dominant phase than that of FeO in ED-MT3 sample. This observation qualitatively suggests that SMS acting as a reducing agent can lower the reduction temperature.

A study [13] reported that at temperatures above 720 °C, Franklinite began to decompose into ZnO and Fe_2_O_3_, as shown in Equation (3). Another important reaction in composite pellets is the Boudouard reaction in Equation (4), where carbon dioxide reacts with solid carbon (C_(s)_) to form carbon monoxide. Compared with solid carbon (coke particles), the contact surface area of gaseous CO with solid EAFD particles is significantly increased, and the reduction speed is accelerated, which is called indirect reduction in pyrometallurgy. ZnO and Fe_2_O_3_ are further reduced by carbon monoxide to Zn and Fe, shown as Equations (5) and (6).

ZnFe_2_O_4(s)_ = ZnO_(s)_ + Fe_2_O_3(s)_ (3)

CO_2(g)_ + C_(s)_ = 2CO_(g)_ (4)

ZnO_(s)_ + CO_(g)_ = Zn_(g)_ + CO_2(g)_ (5)

FeO_(s)_ + CO_(g)_ = Fe_(s)_ +CO_2(g)_ (6)

When the temperature exceeds the boiling point of metallic zinc (907 °C, ΔH = 115.3 KJ/mole), zinc will evaporate and escape from the sample. According to thermodynamic data, at 1100 °C, ZnFe_2_O_4_ decomposed and was reduced into Zn, Fe, and FeO. Carbon can reduce ZnFe_2_O_4_ into gaseous Zn and metal Fe, as indicated in Equation (7), and the reduction occurs at temperatures as low as about ~800 °C [15]. In the industrial Waelz process, zinc is evaporated and recovered as solid oxide (ZnO) by cyclone dust collectors.

ZnFe_2_O_4(s)_ + 2C_(s)_ = Zn_(g)_ + 2Fe_(s)_ + 2CO_2(g)_ ΔG^0^ = 867500–829.4T (J/mole^−1^), when ΔG^0^ = 0, T = 773 °C (7)

The two series of samples shown in Figure 1c (ED-SMS and ED-C) allow a comparison of the kinetics of dezincification effects (or zinc recovery) of different reducing agents. The temperature-dependent Zn removal (D_Zn_, %) of these two series samples were experimentally measured, as shown in the upper curves of Figure 1c. Overall, all samples exhibited an “S-shaped curve”, and the degree of removal can be divided into three parts, namely slow removal ( <15%), rapid increase in removal (15–95%), and complete removal (>95%). At temperature below 800 °C, both series of samples showed relatively low levels of removal, around 10–15%. At temperature above 900 °C, the Zn removal of the ED-SMS samples increased rapidly, reaching approximately 95% at about 1100 °C. On the other hand, the Zn removal of the ED-C samples increased rapidly at temperature above 1050 °C and got nearly 90% above 1250 °C. Finally, the ED-C samples achieved over 98% Zn removal at 1300 °C.

For the series of samples with different torrefaction parameters (ED-M, ED-MT300, and ED-MT500), the effect of torrefaction on Zn removal can be compared with the carbothermic reduction at 800 °C to 1200 °C, and the ED-M (un-torrefied) sample exhibited higher Zn removal than ED-MT3 and ED-MT5 samples (pretreated with torrefaction). The degree of Zn removal of the ED-MT3 sample was slightly higher than that of the ED-MT5 sample, but the difference was negligible. In other words, the main effect of torrefaction on SMS is an increase in fixed carbon ratio. 

In order to quantitatively distinguish the rapid increase (from 15% to 95%) in Zn removal of these two series of samples, the Zn recovery rate (dD_Zn_/dTemp.) can be measured as the derivative of the degree of removal with respect to the temperature, as shown in the lower curves of Figure 1c. The results showed that the EAFD/SMS composite samples had the maximum recovery rate at around 950 °C, while the EAFD/C sample was around 1150 °C. This observation quantitatively suggests that SMS acting as a reducing agent (in place of coke) can lower the reduction temperature by about 200 °C. Therefore, in terms of energy efficiency, the use of SMS as a reducing agent can kinetically accelerate the carbothermic reduction at lower temperatures, thereby reducing energy consumption.

### 3.2. Effect of the Volatile Matter during Carbothermic Reduction

One factor contributing to the superior performance of SMS-based reducing agents compared to coke is the effect of volatile matter (VM). Biomass such as SMS contains large amounts of VM, which may include CO, CO_2_, CH_4_, C_2_H_4_, C_3_H_8_, or other macromolecular hydrocarbons released at temperatures of 300 °C and above [21]. Pyrolysis of macromolecular hydrocarbons begins at 500 °C Equation (8), producing partially-reduced H_2_ [23]. The produced H_2_ promotes the reduction of ZnO and FeO, resulting in H_2_O vapor Equations (9) and (10). 

**Figure 2:**
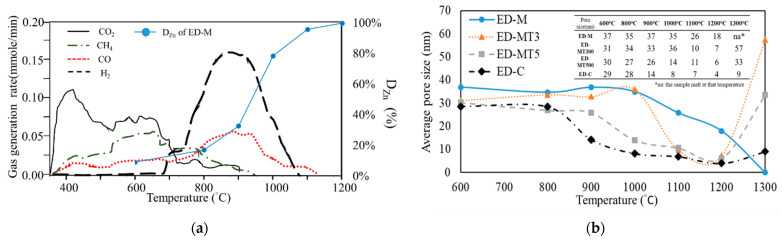
(**a**) Different types of gases vaporized or dissociated from volatile matter (VM) of SMS which was heated from 300 to 1200 °C at the rate of 20 °C/min in inert gas; for comparison, the degree of Zn removal (D_Zn_, %) is also plotted. (**b**) Changes in mean pore size in the carbothermic reduction process from 600 °C to 1300 °C of samples with C/O ratio = 0.8.

Figure 2a showed the gas generation analysis measurement on SMS using a syngas analyzer (MRU-VARIO plus syngas, Germany), followed by heating to 1200 °C at 20 °C/min in inert gas. The small-molecule hydrocarbons are categorized as CH_4_ in Figure 2a, and the macro-molecular hydrocarbons in liquid or solid state (usually called tars or vinegar) are removed by filters to protect the analyzer. Figure 2a showed that above 300 °C, CO_2_ became the main component of the VM, while the content of CH_4_ increased above 500 °C. 

H2 was released in large quantities starting at 650–690 °C, with H_2_ and CO release peaking at 800 °C to 900 °C. It is worth noting that this H_2_ and CO release temperature range (shown in Figure 2a) is the same as the 800 °C to 900 °C range for ZnO reduction (shown in Figure 1c). In this temperature range, the degree of Zinc removal of ED-M samples increased significantly. On the other hand, the similar degree of Zn removal for the ED-C sample (without VM) was at temperatures above 950 °C. Therefore, H_2_ and CO from the SMS VMs play critical roles in carbothermic reduction of ZnO.

(8) $$C_{n}H_{m}=\mathrm{nC}+\frac{m}{2}H_{2(g)}$$

ZnO + H_2(g)_ = Zn_(g)_ + H_2_O_(g)_ (9)

FeO_(s)_ + H_2(g)_ = Fe_(s)_ + H_2_O_(g)_ (10)

It is believed that the release of VM results in the formation of micron to nanometer sized pores in the composite sample pellet. A specific surface area and porosimetry analyzer (Micromeritics Gemin 2360 BET-N2) was used to measure the mean pore size change of samples interrupted at different temperatures during carbothermic reduction. Figure 2b showed an ED-C sample with the mean pore size ranging from 29–30 nm at 600 °C to 800 °C. At higher temperatures from 800 °C to 1200 °C, the mean pore size gradually shrank to below 10 nm. As the temperature increases, more CO_2_ is produced due to the gasification of solid carbon and zinc (gas formed due to the reduction of Franklinite ZnFe2O4, Equation (7)), which is converted into many small pores and, therefore, decreases the mean pore size. Likewise, in the temperature range from 800 to 1100 °C, the mean pore sizes of the SMS-based samples also decreased gradually, as shown in Figure 2b, but the average pore size is larger than that of ED-C sample.

Two types of gases are believed to form in this temperature range, namely VM (Equations (8) and (9)), and the gas formed due to the reduction of Franklinite (Equation (7)). As shown in Figure 2b, the vaporization of VM may lead to the creation of larger pores in the temperature range of 900 °C −1000 °C in high VM samples (ED-M and ED-MT3), which in turn enhances the degree of Zn removal, as shown in Figure 1c, where the EAFD/SMS composite sample has the largest reduction rate at about 950 °C. At temperatures above 1200 °C, due to the sintering of un-reacted oxides (e.g., FeO, CaO, MgO, SiO2, etc. as shown in Table 2), smaller pores will gradually congregate and disappear, and large pores will be formed at 1300 °C.

### 3.3. Effect of Torrefaction on Zn Productivity in Samples Containing SMS Reductant 

**Figure 3:**
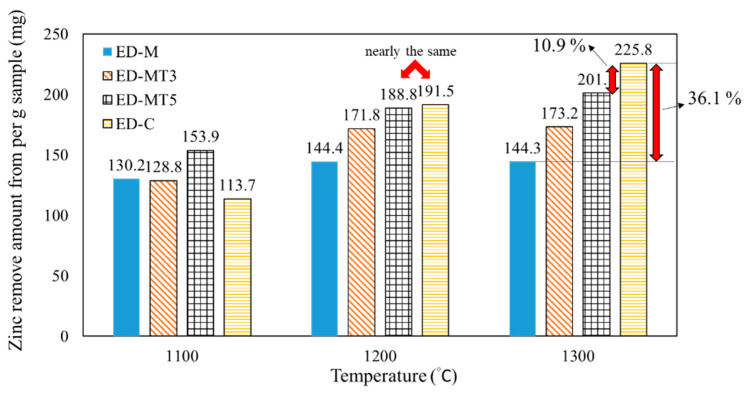
Zn productivity (C/O ratio = 0.8) per gram SMS-based and coke-based pellet samples at different interrupted temperatures (1100 °C, 1200 °C, and 1300 °C) for carbothermic reduction.

**Figure 4:**
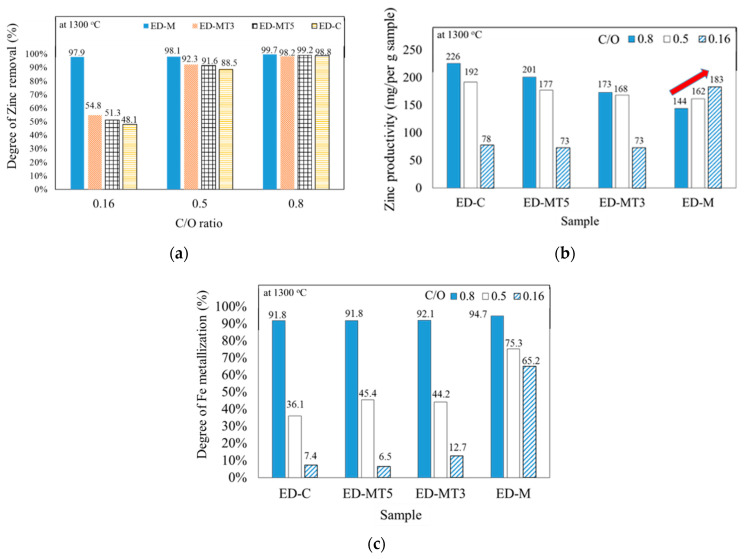
Effect of C/O ratio on the reduction of oxides containing Zn and Fe in EAFD when heated to 1300 °C; (**a**) Degree of Zn removal of samples with different C/O ratios; (**b**) Zn productivity, defined as Zn removal per gram of pellet sample (including EAFD and reducing agent), at different C/O ratios; (**c**) Degree of Fe metallization at different C/O ratios.

For the utilization of biomass carbon, the unit yield of its products is generally lower than that of fossil carbon due to its high water and VM content (or low fixed carbon content). In practice, torrefaction, a technique in which biomass is heated to 300–500 °C in an anaerobic environment to remove moisture and VM, is often used as a pretreatment. The Zn productivity, defined as Zn removal per gram of pellet sample (including EAFD and reducing agent), is shown in Figure 3 and Figure 4b and the following Equation (11).

Zn productivity (mg/per gram sample) = (W_iZn_×D_Zn_)/W_sample_ (11)

where W_sample_ is the weight (g) of pellet sample before the reduction experiment (listed in Table 3). 

Figure 3 compares the Zn productivity of SMS samples torrefied at different temperatures (i.e., un-torrefied, 300 °C, and 500 °C, respectively). As the carbothermic reduction temperature increases, the Zn productivity increases for all samples due to the increased degree of Zn removal. When the temperature reached 1300 °C, the degree of Zn removal (D_Zn_ %) of all samples was above 95%, as shown in Figure 1c.

However, the Zn productivity of the sample without torrefaction (ED-M) was much lower than that of the sample using coke (ED-C). Notably, in Figure 3 of samples reduced at 1300 °C, ED-M sample produced approximately 144 mg of Zn, which is 36% lower than the 226 mg of the ED-C sample. This result is due to the lower fixed carbon content of un-torrefied SMS, which requires the addition of larger amounts of reducing agent if a specific C/O ratio is to be achieved. For torrefied SMS, the VM and moisture contents of SMS-containing samples decreased and the fixed carbon content increased, thereby resulting in higher Zn productivity.

Between the un-torrefied ED-M sample and the ED-MT5 sample reduced at 1300 °C, the Zn production increased from 144 mg to 201 mg, only about 10.9% lower than the 226 mg of the ED-C sample. For samples reduced at 1200 °C, the amount of ZnO produced was closer to that of ED-C sample (189 mg versus 192 mg, only 1.4% difference) due to the higher degree of Zn removal in the ED-MT5 sample. Therefore, the use of ED-MT5 samples resulted in improved Zn removal and productivity compared to the less productive ED-M samples. This is an economically viable option if the torrefaction costs are not considered (assuming SMS disposal costs are equal to torrefaction costs).

### 3.4. Effect of C/O ratio on the Carbothermic Reduction

The aforementioned results indicate that the VM in SMS can generate H_2_, which is beneficial to the progression of the reduction reaction. Therefore, using SMS as a reducing agent can theoretically decrease the consumption of reducing agent and the CO_2_ emissions at the same time. Typically, the amount of reducing agent required stoichiometrically is determined by the molar ratio of reducing agent to oxide or C/O ratio. 

In this study, three C/O ratios were investigated: 0.16, 0.5, and 0.8, respectively. For a C/O ratio of 0.16, it is assumed that ZnO is ultimately reduced by C to become Zn and CO2 according to the reduction Equation (5). Under this assumption, 0.16 is a C/O ratio that is barely sufficient to completely reduce ZnO (about 32.5% of EAFD in Table 2). Likewise, according to the reduction equation (7), a C/O ratio of 0.5 is barely sufficient to completely reduce ZnFe2O4(s). A C/O ratio of 0.8 is typically used in practical settings, which is 1.5 times the minimum C/O ratio for the complete reduction of ZnO and Fe_2_O_3_. This ratio was chosen because, in practice, partially oxidized carbon turns into CO and escapes instead of forming CO_2_.

Figure 4a compares the degree of Zn removal at 1300 °C for samples with different C/O ratios. At a C/O ratio of 0.8, the degree of Zn removal was close to 100% for all samples, indicating that all samples contained sufficient or more than sufficient amount of reducing agent to reduce ZnO. At a C/O ratio of 0.5, all samples showed a slight decrease in the degree of Zn removal, with the largest decrease in the ED-C sample (from 98.8% to 88.5%, Temp. = 1300 °C). For the ED-MT3 and ED-MT5 samples (torrefied at 300 °C and 500 °C, respectively), the drop was smaller than for the ED-C sample, but the difference between the ED-MT3 and ED-MT5 samples can be ignored. For the ED-M sample (without torrefaction), it was minimally affected and maintained a high Zn removal of 98.1%. These results indicate that a C/O ratio of 0.5 is sufficient to reduce most of the ZnO in EAFD.

At a C/O ratio of 0.16, the decrease in the degree of Zn removal was even greater. However, ED-M sample was able to maintain 97.9% Zn removal, much higher than about 50% for all other samples. This indicated that the high VM content in ED-M samples significantly promoted the reduction of ZnO. This high degree of Zn removal at a low C/O ratio suggested that this method can dramatically decrease the consumption of reducing agents and improve Zn recovery rate, thereby increasing crude ZnO productivity.

Figure 4b shows the Zn productivity, defined as Zn remove per gram of pellet sample (including EAFD and reducing agent), at different C/O ratios when heated to 1300 °C. It was observed that the degree of Zn removal decreased with decreasing C/O ratio for all samples except the ED-M sample. Specifically, when the C/O ratio was decreased from 0.8 to 0.16, the Zn productivity in the ED-MT3, ED-MT5, and ED-C samples decreased by 57.8%, 63.6%, and 65.4%, respectively. This drop in Zn productivity is believed to be due to insufficient reducing agents in the pellets.

In contrast, if the C/O ratio was decreased from 0.8 to 0.16, the Zn productivity of ED-M sample increased by 27% (from 144 mg/g pellet to 183 mg/g pellet). This high degree of Zn productivity (close to 98% as shown in Figure 4a) in ED-M sample with a low C/O ratio of 0.16 can be explained by the high percentage VM (over 70% as shown in Table 1) that enhanced the reduction of ZnO. Both ED-M results in Figure 4a,b demonstrate that VM can improve the reduction process, thereby decreasing the amount of fixed carbon required to achieve a high degree of Zn productivity.

In addition to ZnO, EAFD also contains substantial Fe_2_O_3_ (~29%, as shown in Table 2). Currently, EAFD recyclers do not specifically recycle Fe_2_O_3_. Instead, it becomes slag along with other oxides; slag is a waste that requires special handling and carries the risk of secondary pollution. If Fe_2_O_3_ can be reduced and recovered simultaneously with ZnO, it can create added value for EAFD. The amount of slag could also be reduced substantially. 

Figure 4c shows the degree of Fe metallization for samples with different C/O ratios when heated to 1300 °C, indicating that if the C/O ratio decreases, the degree of Fe metallization decreases faster than the degree of Zn removal. For ED-M samples (SMS without torrefaction), Figure 4c shows the degree of Fe metallization decreased from 94.7% to 75.3% and 65.2% as C/O ratio was decreased from 0.8 to 0.5 and 0.16, respectively. In contrast, if the C/O ratio was decreased from 0.8 to 0.16, the degree of Fe metallization of ED-MT3, ED-MT5, and ED-C samples decreased substantially from over 90% to less or close to 10%. 

According to literature reports, the reduction of ZnO starts at around 800 °C, which is lower than that of Fe_2_O_3_ at around 1000 °C, which means that C reduces Zn first. If the amount of C is insufficient, this phenomenon will significantly affect the reduction of Fe_2_O_3_ (as shown by the ED-MT3, ED-MT5, and ED-C samples with C/O less than 0.5 in Figure 4c). Nevertheless, the above results also indicated that the ED-M samples with high VM content were able to maintain the relatively favorable degree of Fe metallization (approximately 65% with C/O = 0.16) promoted by VM. However, the recycling of Fe requires a C/O ratio of 0.8 to achieve high degree of Fe metallization over 90%. If the simultaneous recycling of Zn and Fe in EAFD is to be considered, ED-MT5 with C/O = 0.8 is recommended to achieve a high reduction degree and high productivity of Zn and Fe.

### 3.5. Material Flow Analysis of SMS as Reductant for EAFD and its Effect on CO_2_ Emissions

**Figure 5:**
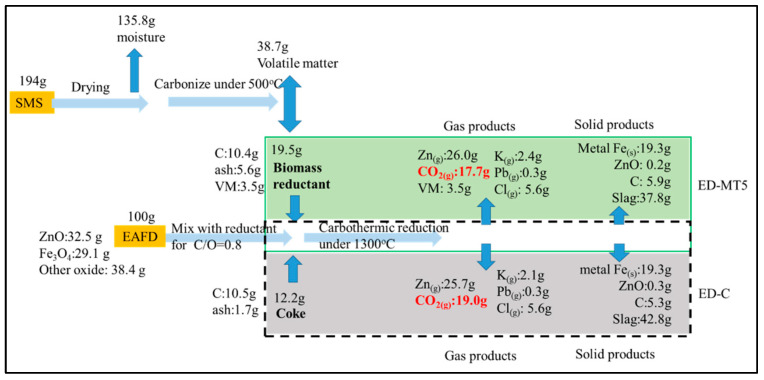
Material flow analysis of ED-MT5 and ED-C as EAFD reducing agents (C/O = 0.8, 1300 °C) and their effects on carbon emissions. Under almost the same Zn products, biomass samples produced less CO_2_ than coke samples. Besides, the biomass reductant showed the potential for extra carbon-reducing according to the assumption of carbon neutrality. Taiwan needs to process 120,000 tons of EAFD annually. If this method is adopted, 23,000 tons of CO_2_ emissions will be reduced, creating a channel to process 230,000 tons of SMS annually.

Figure 5 compares the material flow of coke and SMS (torrefied at 500 °C) as reducing agents mixed with EAFD for reduction. In other words, the mass flow of carbothermic reaction at C/O ratio = 0.8 and 1300 °C and 100g of EAFD were compared for ED-C and ED-MT5 samples, respectively. Initially, the SMS with a moisture content of ~70% weighed 194 g. After dehydration, 135.8 g of moisture was removed. Following torrefaction at 500 °C, most of the VM (38.7 g) was removed. The remaining 19.5 g was used as a reducing agent.

The aforementioned results (Figure 4) show that at C/O = 0.8 and 1300 °C for both ED-MT5 and ED-C samples, the degree of Zn removal will be greater than 98% and the degree of Fe metallization will reach 91%. Therefore, around 26 g of gaseous Zn and 19.4 g of metallic Fe will be produced for both ED-MT5 and ED-C samples. Assuming that all carbon in SMS participates in the carbothermic reduction and becomes CO_2_, then 17.7 g of CO_2_ will be produced. Likewise, the use of coke as a reducing agent yields 19.0 g of CO_2_ to produce the same amounts of gaseous Zn and metallic Fe. The difference in CO_2_ emissions is due to the remaining VM (H_2_) in the SMS. The reduction of H_2_ produces H_2_O, according to Equations (9) and (10). Since SMS belongs to biomass and is carbon-reducing material, the use of SMS as a reducing agent can not only reduce CO2 emissions, but also further consider zero net carbon emissions according to the assumption of carbon neutrality.

With the ED-MT5, CO_2_ emissions can be decreased by 19.0 g, consuming (or recovering) 194 g of SMS that would otherwise be difficult to dispose of (or require additional cost). Taking SMS and EAFD in Taiwan as an example, Taiwan needs to process 120,000 tons of EAFD annually. If this method is adopted, 23,000 tons of CO_2_ emissions will be reduced, creating a channel to process 230,000 tons of SMS annually.

## 4. Conclusions

SMS recycling is a global issue because mushroom crops are grown worldwide. Replacing metallurgical coke with SMS as a reducing agent for EAFD recovery of Zn is a feasible and high value-added SMS recovery method. Besides, the benefits of CO_2_ emission reduction are also in line with the current policy direction of carbon reduction in the world. From this study, the following results are obtained: 

Compared to coke, SMS as a reducing agent lowered the reduction temperature by about 150 °C (from 1250 °C to 1100 °C), resulting in around 95% Zinc removal because H_2_ in the VM of SMS was assisting the reduction. Furthermore, under the same C/O ratio conditions, the torrefaction of SMS increased the productivity (or recovery efficiency) of Zn. The un-torrefied SMS could reduce the amount of reducing agents with the assistance of VM, and the degree of Zinc removal can reach 98% under the condition of C/O = 0.16. Finally, the use of SMS as a reducing agent can reduce CO_2_ emissions because SMS belonging to biomass is carbon-reducing material.

## Figures and Tables

**Table 1:** Results of approximate analysis for SMS (mass %).

Reducing Agents	Fixed Carbon	Volatile Matter (VM)	Ash
SMS *	17.9	72.5	9.5
SMS with T300 °C *	44.1	39.0	16.1
SMS with T500 °C *	59.0	20.3	20.8
Coke	86.9	~0	14.1

* SMS = un-torrefied; T300 °C = Torrefied at 300 °C; T500 °C = Torrefied at 500 °C.

**Table 2:** Results of ash composition for SMS (mass %).

	CaO	MgO	Al_2_O_3_	SiO_2_	K_2_O	Na_2_O	P_2_O_5_	MnO	Fe_2_O_3_
SMS	43.2	6.6	2.8	25.9	6.0	1.2	14.1	0.2	0.8
Coke	8.2	0.6	25.2	56.3	0.7	0.2	0.6	0.3	7.9

**Table 3:** Components of EAFD (mass %).

	ZnO	Fe_3_O_4_	CaO	MgO	MnO_2_	SiO_2_	Al_2_O_3_	K_2_O	Cr_2_O_3_	PbO	S	P	Cl
EAFD	32.5	29.1	13.5	5.1	4.1	4.3	0.5	2.5	0.3	0.3	1.5	0.6	5.6

**Table 4:** Sample parameters.

Sample	C/O Ratio	Ratio of Raw Material		Weight of the Sample Pellet (g) W_sample_
		EAF Dust (EAFD)	Reducing Agent	
ED-M	0.16	90.1	9.9	3.7
ED-MT3		95.5	4.5	5.4
ED-MT5		96.4	3.6	5.7
ED-C		97.6	2.4	5.8
ED-M	0.5	73.3	26.7	3.4
ED-MT3		87.1	12.9	5.2
ED-MT5		89.6	10.4	5.6
ED-C		93.0	7.1	5.7
ED-M	0.8	63.2	36.8	2.8
ED-MT3		80.9	19.2	4.8
ED-MT5		84.3	15.7	5.5
ED-C		89.2	10.9	5.6

ED-M: EAFD + SMS without torrefaction; ED-MT3: EAFD + SMS torrefied 300 °C; ED-MT5: EAFD + SMS torrefied 500 °C; ED-C: EAFD + coke.

## Data Availability

The data that support the findings of this study are available from the corresponding author, Ingann Chen, upon reasonable request.
